# Editorial: Advancements and strategies in predicting and managing clinical drug-drug interactions

**DOI:** 10.3389/fphar.2026.1881790

**Published:** 2026-06-15

**Authors:** Sandhya Subash, Yurong Lai, Allen Tsang, Chuan Li

**Affiliations:** 1 Division of Transalational and Clinical Pharmacology, Cincinnati Children’s Hospital Medical Center, Cincinnati, OH, United States; 2 Drug Metabolism, Gilead Sciences Inc., Foster City, CA, United States; 3 Department of Internal Medicine, School of Medicine, Wake Forest University, Winston-Salem, NC, United States; 4 Shanghai Institute of Materia Medica, Chinese Academy of Sciences (CAS), Shanghai, China

**Keywords:** biomarker, clinical trial, drug-drug interactions (DDI), PBPK, pharmacogenomics

Drug-drug interactions (DDIs) continue to pose significant challenges in clinical pharmacology, translational medicine, and drug development, especially as therapeutic regimens grow more complex with polypharmacy, environmental factors, and the rapid emergence of new targets and innovative modalities. DDIs can alter drug exposure, resulting in treatment failure or adverse clinical outcomes, especially in patients receiving complex therapies or in high-risk settings such as intensive care, oncology, cardiology, and transplantation. The need for accurate DDI prediction, mechanistic understanding, and proactive management is increasingly urgent. The Research Topic “Advancements and Strategies in Predicting and Managing Clinical Drug-Drug Interactions” brings together original research articles, reviews, and perspectives that collectively highlight the evolving landscape of DDI science. This Research Topic spans mechanistic modeling, endogenous biomarker-informed approaches, therapeutic drug monitoring (TDM), transporter biology, pharmacogenomics, and emerging artificial intelligence (AI)-enabled strategies. Together, 12 contributions published in this Research Topic demonstrate how DDI science is being reshaped by real-world evidence, physiologically based pharmacokinetic (PBPK) modeling, biomarker-informed strategies, and human-augmented AI.

Several studies in the Research Topic focus on integrating TDM and real-world evidence into DDI management. Shi et al. evaluated the concomitant use of nirmatrelvir/ritonavir and voriconazole in patients with COVID-19, demonstrating substantial variability in voriconazole exposure and highlighting the feasibility of TDM-guided coadministration despite established contraindications (Shi et al.). Individuals treated with voriconazole in the setting of nirmatrelvir/ritonavir, and Chinese patients requiring dose adjustment when venetoclax is combined with voriconazole (Shi et al.). Likewise, Wang et al. reported that Chinese patients receiving venetoclax with voriconazole had markedly elevated venetoclax exposure, prolonged cytopenias, and higher hospitalization costs, suggesting that further dose reductions beyond current guideline recommendations may be necessary in specific populations (Wang et al.). Chen et al. similarly demonstrated a strong relationship between venetoclax exposure and hematologic adverse events, reinforcing the importance of concentration-guided dosing strategies when CYP3A inhibitors are coadministered (Chen et al.). Collectively, these studies provide important evidence regarding exposure-safety relationships, which are central to DDI management even when the interaction itself is not the primary endpoint. The venetoclax concentration–toxicity study in acute myeloid leukemia further underscores the importance of concentration monitoring to interpret adverse hematologic events and support individualized dosing.

Mechanistic drug metabolism studies in this Research Topic further strengthen the translational perspective. Strong correlations reported for metoprolol- and solanidine-derived metabolic ratios in a real-world cohort provide evidence that endogenous or probe-based phenotyping can characterize CYP2D6-mediated activity in routine practice (Sarömba et al.). Similarly, Ishida et al. combined experimental transporter data with advanced PBPK modeling to elucidate the regional intestinal absorption of talinolol mediated by P-gp and OATP2B1. Their findings demonstrate how regional transporter expression and intestinal physiology can affect local absorption processes and influence exposure in ways not fully captured by conventional dosing assumptions (Ishida et al.). Together, these studies strengthen the value of combining phenotype-based approaches with transporter and enzyme models to better anticipate clinically meaningful DDIs.

PBPK modeling is a major pillar emerging from this Research Topic and exemplifies how quantitative pharmacology can support DDI prediction before clinical application. Wang et al. developed a PBPK platform integrating 13 commonly used drugs in hematopoietic stem cell transplantation (HSCT), demonstrating that PBPK modeling can identify clinically relevant DDIs and optimize individualized dosing strategies during pre- and early post-HSCT care (Wang et al.). Their work illustrates the utility of mechanistic modeling for high-risk populations exposed to narrow therapeutic index drugs and multidrug regimens. Similarly, Byoun et al. used PBPK modeling to investigate CYP2C19-mediated DDIs involving escitalopram in geriatric populations, revealing that aging physiology and pharmacogenetic variability substantially increase DDI susceptibility (Byoun et al.). This investigation highlights the importance of age-related changes in pharmacokinetics, particularly in special populations where DDI risk may be amplified (Byoun et al.). The aforementioned talinolol modeling demonstrates how PBPK analysis can incorporate regional differences in intestinal uptake and efflux, influencing the complex absorption profiles of talinolol (Ishida et al.).

This Research Topic also recognizes that DDIs are deeply embedded within broader clinical and societal contexts. Bellanca et al. reviewed the effects of smoking and smoking cessation on anti-tuberculosis agents, emphasizing how tobacco-related modulation of CYP enzymes can influence drug metabolism, treatment efficacy, and adverse events (Bellanca et al.). Their work points out the importance of considering environmental exposures, lifestyle factors, and behavioral determinants as contributors to clinically meaningful DDIs. The real-world DDI burden in clinical practice is further illustrated by Ghabesha et al., who reported a high prevalence of potential DDIs among hospitalized patients with acute coronary syndrome, largely driven by cardiovascular polypharmacy (Ghabesha et al.). Both articles advocate for a more contextualized view of DDIs, accounting for patient behavior, physiology, and therapeutic goals rather than relying solely on static interaction lists. These findings reinforce the ongoing need for multidisciplinary medication management and clinical pharmacist involvement in high-risk patient populations.

At the same time, the Research Topic also highlights the increasing complexity of DDIs beyond traditional small-molecule paradigms. Cronin and Yu reviewed the unique challenges associated with evaluating DDIs for antisense oligonucleotides and siRNA drugs (Cronin and Yu). Unlike conventional CYP- or transporter-mediated interactions, RNA therapeutics may alter endogenous RNA interference pathways, compete with the RNAi machinery, and cause pharmacodynamic DDIs that are not adequately captured by current regulatory frameworks (Cronin and Yu). This review offers timely insight into the future direction of DDI evaluation as novel therapeutic modalities expand. In parallel, this Research Topic points toward the future of DDI science through the integration of AI and real-world data. Spanakis et al. proposed a human-augmented, mechanistically informed AI framework for proactive DDI management (Spanakis et al.). Their perspective emphasizes the need to move beyond static rule-based interaction checkers toward dynamic systems that integrate molecular mechanisms, pharmacokinetic modeling, clinical expertise, pharmacovigilance data, and patient-specific contextual information. Such approaches may ultimately enable more personalized, explainable, and adaptive DDI prediction systems. Importantly, AI may improve detection and prioritization, but its greatest value will likely come when it complements, rather than replaces, pharmacokinetic reasoning and clinical judgment, a message that remains important for the field.

Collectively, the 12 articles in this Research Topic demonstrate that DDI science is undergoing a significant transformation, becoming more integrated, quantitative, and patient-centered. Advances in PBPK modeling, transporter and enzyme biology, pharmacogenomics, endogenous biomarkers, TDM, AI-enabled analytics, and real-world evidence generation are reshaping how DDIs are identified, predicted, and managed. Importantly, these studies show that clinically relevant DDIs rarely arise from a single mechanism; instead, they stem from complex interactions among drug properties, physiology, genetics, disease states, environmental exposures, and healthcare systems.

Looking ahead, several key priorities emerge. As precision medicine advances, future DDI research will require integrated, interdisciplinary methods that connect mechanistic pharmacology, systems modeling, translational science, and real-world clinical practice. We aim for this Research Topic to provide readers with a comprehensive overview of recent advances and a forward-looking perspective on opportunities and challenges in enhancing medication safety and treatment effectiveness.

We sincerely thank all authors, reviewers, and editors who contributed to this Research Topic and helped advance the field of clinical DDI research.

